# GEMINI: a computationally-efficient search engine for large gene expression datasets

**DOI:** 10.1186/s12859-016-0934-8

**Published:** 2016-02-24

**Authors:** Timothy DeFreitas, Hachem Saddiki, Patrick Flaherty

**Affiliations:** Computer Science Department, Worcester Polytechnic Institute, 100 Institute Rd, Worcester, 01609 USA; Program in Bioinformatics and Computational Biology, 100 Institute Rd, Worcester, 01609 USA; Biomedical Engineering Department, Worcester Polytechnic Institute, 100 Institute Rd, Worcester, 01609 USA; Department of Mathematics and Statistics, University of Massachusetts, Amherst, 710 N. Pleasant St, Amherst, 01003 USA

**Keywords:** Genomic search, Vantage-point tree, Cancer Genome Atlas

## Abstract

**Background:**

Low-cost DNA sequencing allows organizations to accumulate massive amounts of genomic data and use that data to answer a diverse range of research questions. Presently, users must search for relevant genomic data using a keyword, accession number of meta-data tag. However, in this search paradigm the form of the query – a text-based string – is mismatched with the form of the target – a genomic profile.

**Results:**

To improve access to massive genomic data resources, we have developed a fast search engine, GEMINI, that uses a genomic profile as a query to search for similar genomic profiles. GEMINI implements a nearest-neighbor search algorithm using a vantage-point tree to store a database of *n* profiles and in certain circumstances achieves an $\mathcal {O}(\log n)$ expected query time in the limit. We tested GEMINI on breast and ovarian cancer gene expression data from The Cancer Genome Atlas project and show that it achieves a query time that scales as the logarithm of the number of records in practice on genomic data. In a database with 10^5^ samples, GEMINI identifies the nearest neighbor in 0.05 sec compared to a brute force search time of 0.6 sec.

**Conclusions:**

GEMINI is a fast search engine that uses a query genomic profile to search for similar profiles in a very large genomic database. It enables users to identify similar profiles independent of sample label, data origin or other meta-data information.

**Electronic supplementary material:**

The online version of this article (doi:10.1186/s12859-016-0934-8) contains supplementary material, which is available to authorized users.

## Background

Research labs, sequencing core facilities, hospitals and research consortiums are accumulating massive databases of gene expression and other genomic data from primary patient samples. Currently, the GEO database for microarray data contains more than 800,000 samples [1], the International HapMap 3 Project contains 1.6 million common SNPs for 1184 individuals [2], and the Cancer Genome Atlas Project has 1.059 petabytes of genomic data on more than 20 types of cancer [3]. While these databases are already massive, the low-cost of next-generation sequencing is making it easier to add more data to these repositories and to build massive private data repositories [4]. Samples in these databases are often lightly annotated with clinical information or deidentified entirely for patient privacy. The question we address here is: “When a new patient sample arrives, what other samples, among those that we have seen, are most similar to this new one?” The solution we describe here, GEMINI, is a search engine that provides fast access to relevant samples in a database based only on similarity of gene expression profile, much like the PageRank algorithm provides access to internet web pages based on similarity between query terms and terms used in the web page content [5].

Previous work on search engines for gene expression data largely falls into two categories: those that use a gene set query and those that use an expression profile query. ExpressionBlast takes as input a species type, a gene list, an output species and a distance metric and uses text analysis methods to return labeled relevant experiments [6]. SEEK uses a novel cross-validaton-based algorithm to prioritize ranking and network information to identify relevant neighbors based on a query gene set for human data [7]. GeneChaser is an earlier effort that identifies all experiments where a single gene is differentially expressed [8]. In contrast to gene-set-based query search tools, ProfileChaser uses a GEO accession number query to identify experiments that are similar to query [9]. The focus of that work is on choosing good data representation, dimensionality reduction, and similarity/distance metrics. However, they do not evaluate the computational performance or scalability of their approach. We build on the work in ProfileChaser by focusing on speed and scalability while allowing for different dimensionality reduction methods and distance metrics.

Our focus is on developing a method that is amenable to different data representations, dimensionality reduction methods, and distance metrics, and, importantly, is fast. Tree data structures are common in search applications where optimizing query time is important [10]. By structuring the data records into a tree, a suitable algorithm is able to exclude irrelevant records from consideration and reduce search time to less than the brute force complexity of $\mathcal {O}(n)$, where *n* is the number of records in the database. Some binary tree data structures used for search include kd-trees [11], SR-trees [12], R*-trees [13]. Hash tables have very good search time for finding an exact match, but there is no good way to locate a record that is a nearest neighbor to a query. So, while hash tables are often used in applications where exact matches are needed, they are rarely used in application where near matches are needed.

GEMINI uses a vantage-point tree (vp-tree) data structure to store genomic data records [14, 15]. The vantage-point tree is a special case of a binary search tree where the left subtree of a node contains records that are closer than some distance, *μ*, and the right subtree contains records that are further than *μ*. The tree gets its name because the subtree nodes are partitioned from the vantage point of the current node. The advantage of the vp-tree in genomic search applications lies in the fact that is does not impose a particular coordinate structure on the data and instead employs a user-definable metric to measure distance. The construction and search algorithms for the vp-tree are described in the “Implementation” section.

## Implementation

We describe the algorithms for the construction and search for the vantage-point tree here. GEMINI is implemented in python as a stand-alone command-line program and as a public web site; we describe those implementations in the “Availability and requirements” section.

### Data organization

A record in GEMINI is a normalized gene-expression profile. In the Cancer Genome Atlas project, this profile is a level 3 processed gene expression tab-delimited file. These records are converted to a HDF5 file format for compatibility and then preprocessed into a vantage-point tree. Internally, each tab-delimited file from the TCGA project consists of a vector of gene identifiers (e.g. “BRCA1”), and a vector of sample identifiers (e.g. “TCGA-59-2349...”), along with a matrix of the log2 normalized expression value for each gene-sample pair. A query is likewise an HDF5 file with the same attributes but with only one sample. A search therefore returns the most similar expression profiles in a dataset to the profile in the query.

The vantage point tree is implemented as a python class and is entirely loaded into RAM. For datasets with thousands or millions of samples of complete gene expression profiles, the object requires several gigabytes of memory. Though memory performance is somewhat system-dependent, in our tests a database of 100,000 records required 4GB. By reducing the complexity of the profiles using principal component analysis (PCA), the memory footprint can be reduced.

### Vantage-point tree construction

Construction of the vp-tree takes $\mathcal {O}(n \log n)$ time for records with constant dimension where *n* is the number of records in the dataset. We briefly summarize the simplest version of the recursive construction algorithm here and refer to the original article for further details and extensions [14].



This binary search tree construction works by taking a set $\mathcal {S}$ of records. If $\mathcal {S}$ is not empty, we create a new node and store a random element, *p*, in the node. We store the median distance between *p* and all the other elements in $\mathcal {S}$ in *μ* in the node using any distance metric that satisfies the triangle inequality. We partition the set $\mathcal {S}$ into two roughly equal size sets L and R, where L contains all of the elements of $\mathcal {S}$ that are closer to *p* than the median distance, *μ* and R contains all of the elements of $\mathcal {S}$ that are further than *μ*. The function recurses by calling itself with arguments L and R for the left (closer) and right (further) subtrees. The recursion ends when the subtree sets are empty and the algorithm returns the pointer to the root node. Clearly, because the size of the set in each subtree is half the original set, due to the use of the median distance, the time to construct the tree is $\mathcal {O}(n \log n)$.

### Vantage-point tree search

Search in the vantage-point tree proceeds by recursive depth-first search. The left subtree of a node contains records that are closer than *μ* from the vantage point of the current node’s records. Symmetrically, the right subtree contains records further than *μ*.

If we have a query profile, *q* and a vantage-point node, *p*, by symmetry and the triangle inequality of a distance metric *d*(·,·), we have

(1) $$ d(q,s) \geq | d(q,p) - d(p,s) | = d_{p}(q,s),  $$

where *s* is any other record in the database and *d*_*p*_(·,·) is defined as the vantage-point distance. Since the vantage-point distance shrinks the true distance between *q* and *s*, if *d*_*p*_(*q*,*s*)≥*τ*, then *d*(*q*,*s*)≥*τ* [14].

**Fig. 1:**
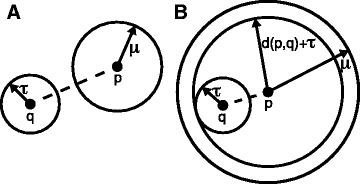
Vantage-point tree structure allows search algorithm to fathom subtrees. Given we have already found a record at distance *τ* from the query node *q* and we are at vantage point *p* with a right subtree containing records further than *μ* from *p* and a left subtree with records closer than *μ*. **a** If *d*(*p*,*q*)≥*τ*+*μ*, then the nearest-neighbor is not closer than *μ* and we can fathom the left subtree containing records closer than *μ*. **b** If *d*(*p*,*q*)+*τ*≤*μ*, then the nearest-neighbor is certainly closer than *μ* and we can fathom the right subtree

Suppose that we have found a record at distance *τ* from the query and we are at vantage-point node *p* in the tree. If *d*(*p*,*q*)≥*τ*+*μ*, then the nearest-neighbor is not closer than *μ* and we can fathom (remove from further consideration) the left subtree as shown in Fig. 1a. Conversely, if *d*(*p*,*q*)+*τ*≤*μ*, then the nearest-neighbor is certainly closer than *μ* and we can fathom the right subtree (Fig. 1b). Thus, the vantage-point tree data structure allows us to exclude records from examination and we achieve super-linear search time. As shown by Yianilos, the average-case querying time scales as $\mathcal {O}(\log n)$ when the data is low-dimensional [14]. We have found that we achieve good performance if the dimension of the data loaded into the vp-tree is less than 30 and the query time is roughly two orders of magnitude faster than brute force for a dimension of 10 (Additional file 1: Figures S1 and 2).

The search algorithm can be written as a recursive depth-first search algorithm as described previously [14]. The algorithm holds the node of the nearest neighbor in the global variable best and is initialized with *τ*←0. If d(q, node) <*μ*+*τ*, only the left subtree is traversed and if d(q, node) >*μ*−*τ* then only the right subtree is traversed. If *μ*−*τ*≤d(q, node)≥*μ*+*τ* then both subtrees are traversed.



Vantage point tree structures support any distance metric that satisfies the triangle inequality, but the optimal distance function is not yet known. For simplicity, GEMINI currently uses the euclidean distance between samples after principal component transformation.

However, weighted distance functions utilizing genomic knowledge could better facilitate a particular search. For example, for a cancer dataset, one could limit the genes compared to known oncogenes, thereby finding which sample showed the most similar oncogenic profile to the query. This search should be more sensitive to small changes in particular genes, and and therefore result in less statistical noise, though we do not attempt to prove this in this paper.

## Results

### Comparison with brute-force and KD-tree methods

**Fig. 2:**
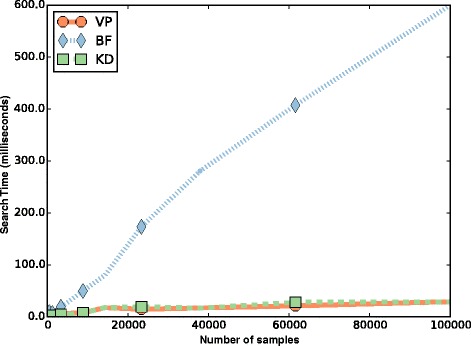
Timing comparison of GEMINI and other search methods. The search time in milliseconds is shown for a typical query in databases ranging in size from 10 samples to 100,000 samples. The plot compares the vp-tree (VP), KD-tree (KD) and brute force (BF) methods. The brute force search time scales linearly with the size of the database, while GEMINI search time scales as the log of the size of the database

We compare the vp-tree-based nearest neighbor search to a KD-Tree and brute-force approach in Fig. 2. We focus exclusively on the speed of the methods since all three algorithms implement the nearest neighbor search algorithm and must return the same list of nearest-neighbor results. The brute force algorithm simply compares the query to every record in the database. As expected, the brute force approach scales linearly in the size of the database. The tree structure methods scale as the log of the size of the database due to the ability of the search algorithm to exclude distant samples from consideration based on their position in the vp-tree.

Though both of the tree-based algorithms scale similarly with the log of the database size in the average case for low-dimensional data, they differ in their construction algorithms. KD-Trees use non-leaf nodes to divide the dataset using a hyperplanes whose normal vector is equivalent to one of the dimensions of the data. Splits continue recursively until the number of instances in each node is smaller than some threshold. Yianilos showed that query time for both the KD-tree and vp-tree scales exponentially with the dimension of the data set (Figure 6 in [14]). Therefore, for both methods, it is important to perform some form of dimensionality reduction prior to storing the data in the data structure.

Both KD-tree and vp-tree are constructed in $\mathcal {O}(n \log n)$, but KD-trees use a sliding midpoint median implementation, while the vp-tree uses a standard linear time median-finding algorithm. Other trees achieve similar complexity and differ in the use of split heuristics and amount of reinsertion during construction.

### Search in Cancer Genome Atlas database

**Fig. 3:**
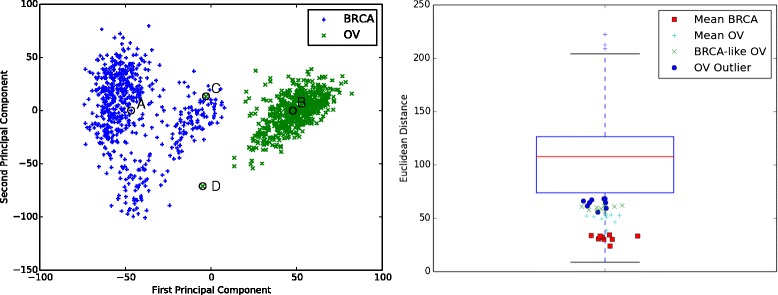
Differential gene expression for ovarian (OV) and breast (BRCA) cancer samples from TCGA. (*left*) Principal component analysis is used to project the 17,813 dimension gene expression data to two dimensions for visualization. The ovarian samples and breast samples clearly cluster. One ovarian sample (*C*) has an expression pattern similar to breast cancer samples and one (*D*) shows an expression pattern outside of both the ovarian and breast clusters. Representative breast (*A*) and ovarian (*B*) samples are circled. (*right*) A boxplot of all non-zero pairwise distances in the joint breast and ovarian cancer data sets. The nearest neighbors for the four queries are shown as symbols in the legend. We find that the nearest neighbors all fall closer than the lower quartile of all of the distances

We tested GEMINI on a database of gene expression data from The Cancer Genome Atlas (TCGA) comprised of 559 ovarian (OV) and 599 breast cancer (BRCA) samples. First, we projected the probe-level data onto the first 10 principal components to reduce the dimensionality of the data from 17,813 features. The choice of 10 was selected due to diminishing returns on the retained variance in the data associated with each subsequent dimension. Using the BRCA and OV dataset, the first 10 dimensions preserved 40 % of the variance in the data, while 4 dimensions preserved just 25 % and 100 more were required to achieve 70 %. A plot of the first two principal components for the BRCA and OV samples is shown in Fig. 3. Clearly, the two cancer types differ in their gene expression patterns and cluster. However, there are two ovarian samples that do not cluster with the rest. One falls within a group of BRCA samples and the other falls outside of either cluster.

**Fig. 4:**
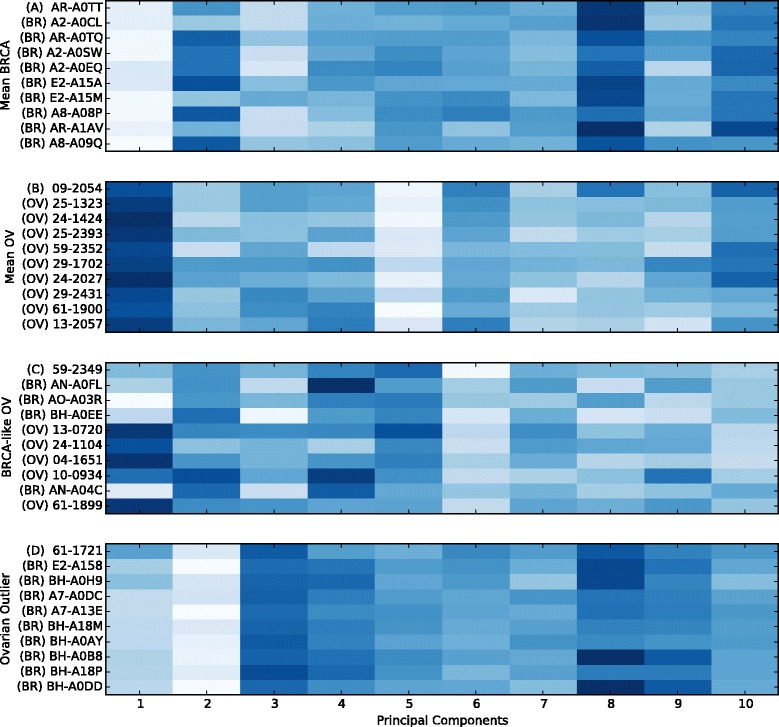
GEMINI heat map results showing 9 nearest neighbors to the query (*top row*) for four samples. Four query profiles were used to search for nearest-neighbor profiles in a database containing both ovarian and breast cancer samples. The nearest neighbors of the prototypical breast cancer profile are all breast cancer samples and the nearest neighbors of the prototypical ovarian cancer profile are all ovarian cancer samples as expected. The ovarian cancer sample that falls in the breast cancer cluster is nearest neighbors with both ovarian and breast cancer samples. The ovarian cancer outlier has all breast cancer samples as nearest neighbors indicating that the differential gene expression patterns for that sample most closely resemble breast cancer

We tested GEMINI using four queries against the database of combined OV and BRCA samples. The four queries (shown circled in Fig. 3) are: (A) a prototypical BRCA sample, (B) a prototypical OV sample, (C) a BRCA-like OV sample, and (D) an outlier OV sample. The prototypical OV and BRCA sample is the nearest Euclidean neighbor to the average OV and BRCA expression respectively. The top 10 hits by similarity to the prototypical OV sample are all OV samples and the top 10 hits for the prototypical BRCA are all BRCA samples as expected (Fig. 4). The BRCA-like OV sample (59-2349) has 4 BRCA samples and 5 OV samples in the top 9 hits. This result indicates that the BRCA-like OV gene-expression pattern has similarity to samples from both BRCA and OV. The OV outlier, surprisingly, shows the most similarity to 9 BRCA samples.

There may be many reasons for the similarity of one gene-expression profile to another. There could be batch effects or other forms of confounding that suggest biological similarity when the true reason is technical artifact. Furthermore, one cannot rule out random expression noise as a cause for similarity based on the results of one query. Validation through experimental means would be required to support true biological similarity. The purpose of GEMINI, instead, is simply to quickly return the nearest-neighbors to a query profile from a large database.

## Discussion

GEMINI forms the basis of an open-source platform for machine learning optimization of search result relevance in genomic data repositories. By observing the click-through behavior of an individual or group of users, the platform may learn and re-rank results based on individualized probabilistic assessments of relevance.

This search engine fits in the context of a large database of profiles that are centrally located as well as with distributed databases. While it may be impossible to store all of the public genomic data in one repository, autonomous software that crawls the web identifying genomic data resources can temporarily store the profile long enough to identify the insertion location in the tree. Only the url of the root source of the data would then be needed in the vp-tree. Then, if the record is identified as a near-neighbor, the profile can be retrieved on-demand.

Our capability to generate genomic data is outpacing our capability to analyze and re-use that data. A fast, accurate search engine for genomic data may enable researchers to make discoveries using community-collected data more effectively. GEMINI uses a vp-tree to enable us to make effective use of the massive genomic data repositories.

## Conclusions

Current genomic data search engines use text-based queries to search for numerical (e.g. gene expression) genomic data profiles. But this paradigm represents a mismatch between the subject and object of the query. Our genomic data search engine, GEMINI, matches the query and database record forms and leverages a vp-tree data structure to find the nearest-neighbor records in a gene-expression database with a focus on search speed.

## Availability and requirements

We have implemented GEMINI as a python module, a standalone command-line program and as a website. Our code extends an implementation of the vp-tree originally written by Huy Nguyen whose code is available [16]. Paul Harrison’s code was also very helpful for our implementation [17]. The KD-tree was implemented using a scipy library written by Anne Archibald [18]. Usage documentation for the python module is provided with the source code.

The standalone command-line program has two sub-commands: build and search. The build sub-command takes a HDF5 format file with three datasets: “Sample”, “Feature”, “Data” and returns a pickled vp-tree data structure. The source data contains sample names in “Sample”, genomic features names (genes) in “Features” and the data matrix (features x samples) in “Data”. The search sub-command loads the vp-tree structure created in the build step and a HDF5 file in the same format as the source data except with a single column for the “Data” vector as the query. GEMINI prints the top K matches in the source data matrix where K is 10 by default but can be modified in command-line options.

The web interface at genomics.wpi.edu/gemini has only one entry box for the user to specify the query HDF5 or CSV file. The vp-tree is built off-line and loaded using a separate administrative tool and associated with a specific query page for the data source. This design choice provides a robust and simple interface and minimizes the user-effort to search. After submitting the query, the user is directed to a results page that shows a heatmap representation of the top 10 matches to the query.

**Project name:** GEMINI**Project home page:**http://genomics.wpi.edu/gemini**Operating system:** platform independent**Other requirements:** python modules listed in requirements.txt on version control site (https://bitbucket.org/flahertylab/gemini). None for website.**License:** Creative Commons Attribution 4.0 International(http://creativecommons.org/licenses/by/4.0/)

## Additional file

Additional file 1
**Supplementary information.** (PDF 81 KB)
