# Supplementary material for: GEMINI: a computationally-efficient search engine for large gene expression datasets
Source: BMC Bioinformatics. 2016 Feb 24;17:102. doi: 10.1186/s12859-016-0934-8 (PMC4765211; doi:10.1186/s12859-016-0934-8)
Supplement: Additional file 1 — Supplementary information. (PDF 81 KB) [file 12859_2016_934_MOESM1_ESM.pdf]

Supplementary Information

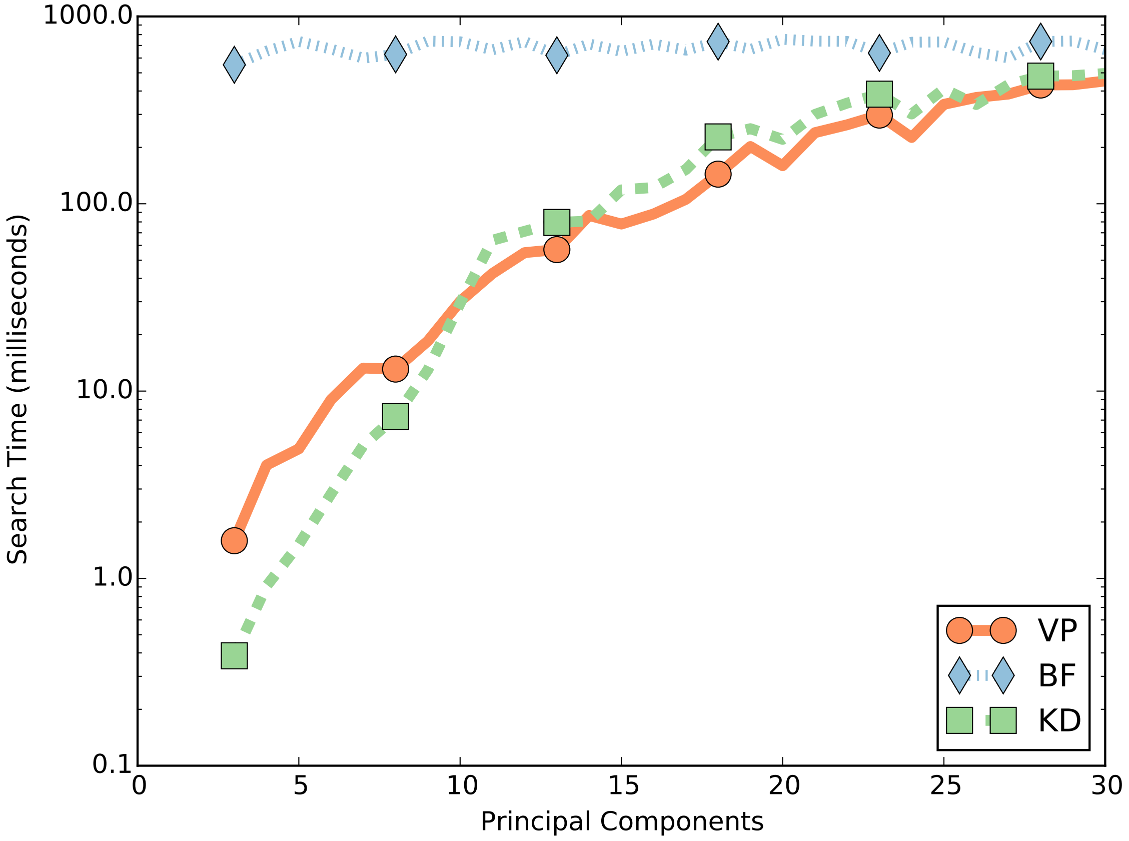

Figure 1: Relation between query time and data dimension for vp-tree, KD tree, and brute force methods. The vp-tree and KD-tree methods are much faster than the brute force method for data dimensions less than 10. As the dimension of the data increases towards 30, the search time approaches the brute force method. Search times are an average of 5 queries identifying the top 3 nearest neighbors against a Gaussian random simulation data set with sample size 100,000 reduced by principal component analysis.

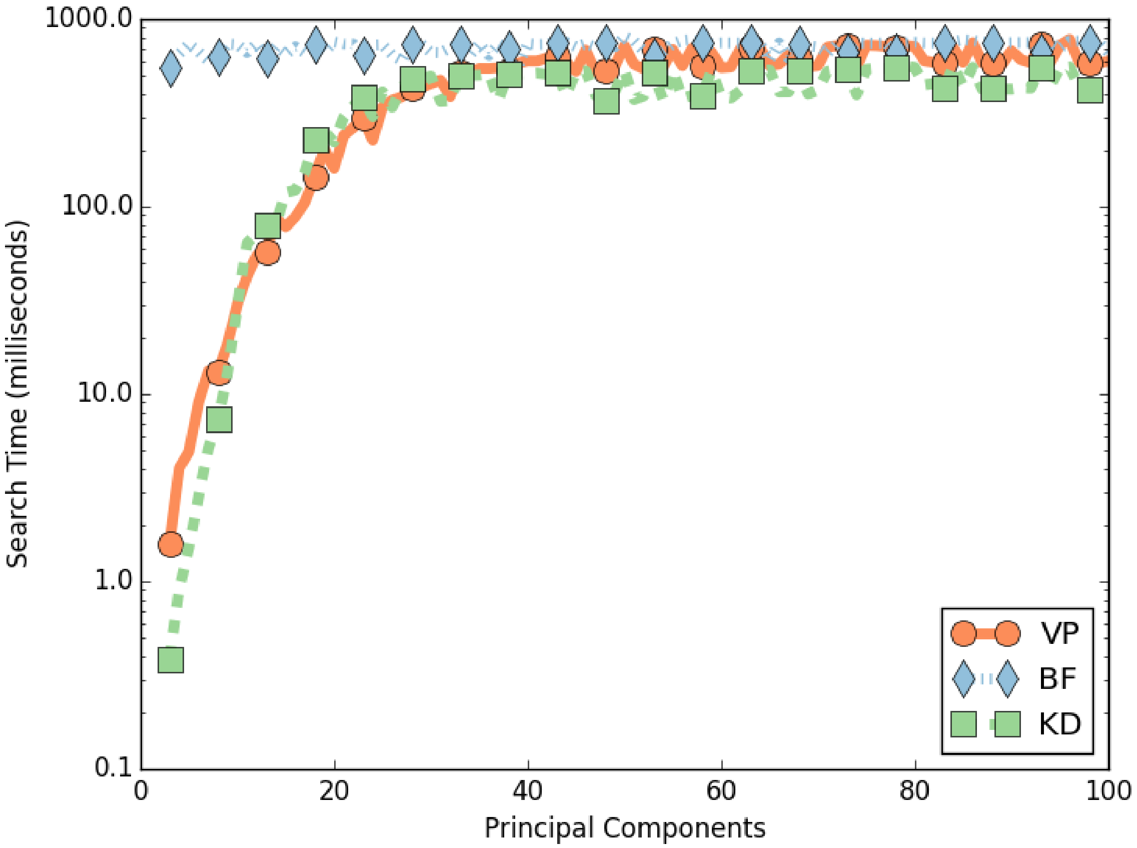

Figure 2: Relation between query time and data dimension for vp-tree, KD tree, and brute force methods. The search time asymptotically approaches the brute force time as the number of dimensions increases. Search times are an average of 5 queries identifying the top 3 nearest neighbors against a Gaussian random simulation data set of sample size 100,000 reduced by principal component analysis.
